# Evolution of natural disaster terminologies, with a case study of the covid-19 pandemic

**DOI:** 10.1038/s41598-024-64736-8

**Published:** 2024-06-25

**Authors:** H. Jithamala Caldera, S. C. Wirasinghe

**Affiliations:** Department of Civil Engineering, University of Calgary, Calgary, AB Canada

**Keywords:** Disaster, Catastrophe, Emergency, Disaster terminologies, Disaster categorization, Severity scale, Natural hazards, Environmental impact, Climate-change impacts, Environmental impact, Climate change

## Abstract

Disaster, catastrophe, and cataclysm are some English terminologies that describe the severity of adverse events. Civilians, reporters, and professionals often use these terminologies to communicate and report any event’s severity. This linguistic method is the most practical way to rapidly reach all levels of local/regional/national, and international stakeholders during disasters. Therefore, disaster terminologies play a significant role in disaster management. However, attaining the actual magnitude of a disaster’s severity cannot be comprehended simply by using these terminologies because they are used interchangeably. Unfortunately, there is no consistent method to differentiate disaster terminologies from one another. Additionally, no globally accepted standard technique exists to communicate the severity level when disasters strike; one observer’s ‘disaster’ can be another’s ‘catastrophe’. Hence, a nation’s ability to manage extreme events is difficult when there are no agreed terminologies among emergency management systems. A standard severity classification system is required to understand, communicate, report, and educate stakeholders. This paper presents perceptions of people about disaster terminologies in different geographical regions, rankings and differences in disaster lexical and lexicon. It explores how people perceive major events (e.g., the Covid-19 pandemic), and proposes a ranking of disaster terminologies to create a severity classification system suitable for global use.

## Introduction

The linguistic method, which involves using generic terminology such as ‘emergency,’ ‘disaster,’ and ‘catastrophe’, plays a vital role in communicating the severity of natural disasters—events triggered by nature, such as biological, climatological, extraterrestrial, geophysical, hydrological, and meteorological disasters. Within a language community, terminologies serve to convey information and inter-subjective messages. Like all forms of written or oral communication, the meaning of a term (or sentence) carries a message and information from one person to another. The linguistic method represents the oldest and most practical method of communication and reporting during disasters of varying severity levels. It rapidly disseminates information to all levels of stakeholder groups (local, provincial, regional, national, continental, or international). Additionally, it is commonly employed for educational purposes due to its wide-reaching effectiveness.

The need for a common understanding of terminologies to communicate about the severity of an event is essential in disaster situations^1^, as they often require many stakeholders to work together toward a common goal. This need is compounded by the fact that many stakeholders involved in disaster relief may speak different languages and might not fully understand English words such as ‘disaster,’ ‘catastrophe,’ and ‘cataclysm.’ As a result, confusion and breakdowns in communication may occur because some stakeholders might not comprehend the exact meaning of the terminologies being used^2^. Yet, establishing a common understanding of these terminologies is vital to reduce confusion among stakeholders and create a well-understood framework for providing disaster relief. However, for this to happen, policymakers, academics, and practitioners must initiate the process of redefining terminology while simultaneously developing appropriate measures and scales that distinguish each term and its representation of disaster severity.

Similarly, although disasters are not universally understood in the same way^3^, reaching a common understanding—such as a universally agreed-upon approach about what category a disaster falls under–to classify disaster severity is essential^4^ in disaster management and disaster risk management. This common understanding is crucial in situations where many stakeholders worldwide come together for a common cause. To initiate this process, English is the most suitable language for classifying natural disasters globally, given its predominance as the most widely spoken and used official language (see Supplementary A online). The disaster terminologies in English are even adapted with modifications when creating severity scales (for both individual and common classification systems, as shown in Supplementary B online) to emphasize the degree of impact an event has^5^. When an acceptable point of reference exists within a globalized language (i.e., a guideline or standard criterion) to classify disasters, it can be adapted to any particular language through translation and standard colour and number coding, as described in Section “Limitations and future extensions”. 

Due to inconsistency in how stakeholders perceive various terminologies, the lack of agreed terminology poses a global challenge in formulating legislation and policies regarding disaster response^6^. Failing to identify a potential hazard during disaster communication can lead to devastating consequences^7^. For example, Hurricane Katrina’s devastating impacts were exacerbated by an ineffective government response and a failure to recognize the severity of the situation^8^. Another example is the series of decision-making errors that compounded disaster relief efforts during the 2004 Indian Ocean tsunami. These errors contributed to the Indian Ocean Tsunami becoming one of the world’s deadliest natural disasters, resulting in approximately 230,000 deaths and leaving over a million homeless in 14 countries. For instance, in India, word of the disaster went to the wrong official^9^. With no warning, coastal populations were caught off guard by the immense waves from the tsunami. The lack of communication was made worse because officials did not recognize or adequately classify the severity of the event. Consequently, coastal residents, tourists, and governments did not know the tsunami’s severity, so they did not effectively respond to the disaster^10^. In addition, inconsistent identification of disaster impacts results in overcompensation or under-compensation in assigning resources for mitigation^11^. Overcompensation may waste resources, while under-compensation could increase the impact severity. Properly and promptly identifying the disaster impact is crucial because lives depend on these decisions^12^.

These examples make it imperative that a standard severity classification system is required to understand, communicate, report, and educate stakeholders during a disaster, including in both the pre- and post-disaster period. Moreover, these natural phenomena have no national boundaries when they strike. The impacts of a disaster in a region, if not managed properly, can produce political and social instability, and affect international security and relations^13^. The recent Covid-19 pandemic is a good example of these consequences. A common communication platform for disaster severity is therefore needed to convey vital information in a standard format that a global audience understands.

Selecting specific terminologies, even within the English language, to represent varying levels of severity for a global audience is a challenging task that demands careful consideration. This challenge arises because the terminology we employ is not universally understood. The lexicon (dictionary) meaning and the lexical (verbal) meaning of these terminologies can vary based on factors such as time/era, location, individual experience, and situation (see Supplementary C online). Although the severity scales mentioned in Supplementary B online are developed using the linguistic method to categorize the different levels of severity, in all but the two common scales, the proposed labeling appears to be arbitrary, particularly in all individual and common scales. The two scales, Fatality-Based Disaster Scale^14^ and Universal Disaster Severity Classification^15^, paid some attention when selecting the terminologies to label the levels of severity; however, they did not consider the current views of the people who are going to use these scales. Proposing a clear definition and criteria for disaster terminologies is important, but people often do not refer to the definition, especially in disaster situations, and they assume the lexical (verbal) meaning of the word. Therefore, it is important to consider the users’ perspectives when selecting labels/terminologies for unification to categorize disaster severity levels.

This research aims to propose a universal classification framework for defining disaster severity regardless of the geographical location of the disaster and the linguistic, lexical, and semantic nuances that can affect the interpretation of terminology. Moreover, the focus of this universal disaster framework will be measured specifically in terms of the adverse effects the event has on a community or an environment and not the degree of severity it has on an individual.

## Methodology

The terminologies that describe the magnitude of a natural phenomenon, including calamity, cataclysm, catastrophe, disaster, and emergency, were selected for investigation. The aim was to determine whether significant differences exist in the seriousness levels among these terms or if people perceive them as synonyms and use them randomly. The term ‘Armageddon,’ which describes “a usually vast decisive conflict or confrontation” or “a terrible war that could destroy the world”^16^, was excluded from consideration due to its relevance to human-caused catastrophes rather than natural events. Similarly, the term ‘apocalypse’ was excluded from the analysis due to its religious connotations, as some religious beliefs associate it with the destruction or end of the world.

Surveys have been conducted to investigate people’s perceptions of natural disaster terminologies and how they utilize these terms to indicate the severity levels of an event using a case study. The primary objective of these surveys was to determine whether differences exist in ranking disaster terminologies among individuals. The research question addressed was: Are there any differences in the ranking of disaster terminologies among people? The hypothesis posited is that there are no differences in the ranking of disaster terminologies among people. Therefore, the independent variables in this study were the disaster terminologies, and the dependent variable was the respondents’ rankings of the disaster terminologies. These surveys were approved by the University of Calgary Conjoint Faculties Research Ethics Board.

To examine the previous research question, two web-based surveys were conducted. All five terminologies (calamity, cataclysm, catastrophe, disaster, and emergency) were presented in alphabetical order to each respondent. Subsequently, the respondents were asked to rank the five terminologies based on their understanding of the term’s severity level, ranging from the lowest (Level 1) to the highest (Level 5). Respondents were not allowed to assign the same rank to two different terminologies within these surveys. The first survey, conducted from August 2015 to December 2020, involved presenting the terminologies without providing their definitions, resulting in rankings based on lexical (verbal) meaning. In the second survey (conducted from September 2020 to June 2021), respondents were given the definitions from the Oxford dictionary, resulting in rankings based on lexicon (dictionary) meaning. The study seeks to rank the severity of disaster terminologies for global audiences who typically rely on dictionary definitions rather than disaster literature when referencing meanings. It is noteworthy that none of the disaster literature, except for the literature related to the continuation of this research, provides definitions for all five terms considered in this study. Consequently, the current definitions from the Oxford English Dictionary for the aforementioned five terms are presented to the respondents to ensure consistency in the analysis. During the second survey, respondents were also queried about the single terminology they would use to describe the ongoing Covid-19 pandemic. Additionally, real-time Covid-19 statistics, including global confirmed cases, global deaths, and global recovered cases, were presented to the respondents while answering this question.

Web-based international surveys were conducted to provide access to large and geographically dispersed populations cost-effectively and efficiently. These web surveys were launched on the Alchemer platform (formerly known as SurveyGizmo) to reach participants globally. As this study was conducted in English, the target population comprised English-speaking adults aged 18 years or older who were internet users, amounting to fewer than 1 billion people. Approximately 1.4 billion out of 7.8 billion people spoke English, with around 26% of the global population being under 15 years of age^17^. Additionally, there were 4.914 billion active internet users worldwide, constituting 63% of the global population in 2021^18^. The survey was designed as an international web-based survey due to its focus on the adult English-speaking population (aged 18 and above). While no subgroups were identified within the global population analysis, subgroups were considered for geographical areas, such as the six populated continents. A general statistical guideline suggests that approximately 30 participants are needed in each group^19^. However, the sample size requirement for non-parametric tests was 1.15% of the parametric test’s sample size^20^. Consequently, a sample size of about 242 was necessary to represent all continents in non-parametric tests.

In this study, non-probabilistic sampling techniques are employed because the research focuses on the entire population of English-speaking adult internet users, a group too vast to be comprehensively examined. The study aims to establish a ranking suitable for a global audience, rendering it impractical to utilize random probability sampling, which would grant each population member a known (or equal) chance of participation. Consequently, a combination of convenience sampling (recruiting readily available and willing participants)^19^ and snowball sampling (recruiting participants through existing participants)^19^ was employed to gather respondents. Potential survey participants were invited through both professional and personal networks, including contacts gathered from the 3rd United Nations World Conference on Disaster Risk Reduction. Survey links were disseminated through various means, including emails, short message services, social media platforms (such as Facebook and LinkedIn), newsletters (e.g., the e-PEG newsletter of the Association of Professional Engineers and Geoscientists of Alberta, and the electronic newsletter of the World Federation of Engineering Organizations—Committee on Disaster Risk Management), websites, online discussion platforms (such as Catastrophe Indices and Quantification Inc. (CatIQ), and the Canadian Risk and Hazards Network (CRHNet)), and by distributing handouts at conferences (including the 12th and 13th Annual CRHNet Symposiums, the 5th International Natural Disaster Mitigation Specialty Conference of the Canadian Society for Civil Engineering, the 12th International Conference of the International Institute for Infrastructure Resilience and Reconstruction, and the Canadian Catastrophe Conference of the CatIQ).

Since the study was based on ordinal data (ranking/ordering values)^20^, it was better suited for representation by the median rather than the mean. Consequently, non-parametric tests were employed. The preference for the median over the mean stemmed from the skewed distribution nature of the study. The median better captures the center of the distribution, signifying that 50% of the values lie above it while 50% lie below. For instance, consider a scenario where most individuals assign higher rankings to a particular term, and very few assigns lower rankings (resulting in a few outliers) to that same term. In such cases, the mathematical mean may decrease even though the median remains stable. In situations where the distribution is significantly skewed, extreme values in the distribution’s tail can substantially affect the mean. Conversely, the median remains a more robust indicator of the distribution’s center.

In these surveys, the five samples of each terminology are interconnected, as respondents were unable to assign the same rank to two different terminologies. As a result, the ranks received for the five terminologies were interdependent. The non-parametric tests below^21^ were conducted to address the following hypotheses:

1. The Friedman test was employed to determine whether people utilize these five terminologies randomly or if there exists a significant difference in the ranking of each terminology.
  **H0**
  There is no significant difference between the mean ranks of the disaster terminologies.
2. If the ranks were not randomly distributed, Kendall’s W Test was performed to ascertain the agreement between respondents’ rankings.
  **H0**
  There was no agreement among the respondents in ranking different terminologies (W = 0).
3. In cases where agreement among respondents’ ranking was observed, the Wilcoxon signed-rank test was conducted for each pair of terminologies. This aimed to identify terminologies with differing rankings and terminologies showing similar rankings, shedding light on peoples’ ranking of these natural disaster terminologies. Further details about the Wilcoxon signed-rank test are available in Supplementary D online.
  **H0**
  The median difference (M_A_ - M_B_) was equal to zero. For instance: H_0_: M_Cataclysm_ − M_Calamity_ = 0.

## Analysis of perception about natural disaster terminologies

**Figure 1:**
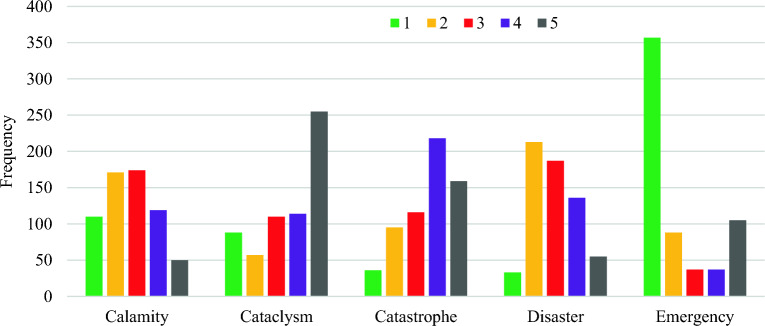
Frequency distribution of respondents’ rankings (from 1 to 5) for natural disaster terminologies.

To gauge public perceptions of commonly used severity terminologies, an initial survey collected 1170 responses. However, only 624 respondents (approximately 54%) completed rankings for all five terminologies based on their lexical (verbal) meanings. Notably, many respondents omitted rankings for ‘cataclysm’ or ‘calamity’ compared to the other three terminologies. ‘Emergency,’ ‘disaster,’ and ‘catastrophe’ were more widely recognized, likely due to their prevalence in governmental and insurance-related contexts, while ‘calamity’ and ‘cataclysm’ were viewed as more colloquial^15^. Some respondents may have refrained from ranking these terminologies due to their perceived subjectivity, favouring a more objective approach to assessing disaster severity. The initial assumption that ‘emergency,’ ‘disaster,’ ‘calamity,’ ‘catastrophe,’ and ‘cataclysm’ represent a perceived hierarchy of seriousness among disaster terminologies was derived from the frequency of completed survey rankings (see Fig. 1).

Based on the two-survey analysis (see Supplementary E online), Table 1 compares respondents’ rankings of disaster terminologies with and without the respective terminology’s definitions. In the global sample, the absence of the terminology’s definitions (i.e., lexical (verbal) meaning) resulted in four distinct levels of ranking for ‘emergency,’ ‘calamity,’ ‘disaster,’ and ‘catastrophe/cataclysm.’ However, when the definitions of the terminologies were provided (i.e., lexicon (dictionary) meaning), respondents did not differentiate between terminologies with more than two levels, specifically ‘emergency/calamity’ and ‘disaster/catastrophe/cataclysm.’ Similarly, for North Americans and Asians, the absence of terminology definitions led to clear differentiation among ranks, creating three distinct levels: ‘emergency,’ ‘calamity/disaster,’ and ‘catastrophe/cataclysm.’ However, with the presence of definitions, they did not differentiate terminologies with more than two levels. Additionally, Oceania respondents, who exhibited two distinct levels without the presence of disaster terminology definitions, showed only one level when definitions were provided (i.e., they randomly ranked the five terminologies). Europeans maintained the same rankings with or without definitions. However, it is important to note that the European and Oceania continents might not have yielded accurate results due to insufficient data (n < 60) for the Wilcoxon signed-rank test. Nevertheless, a clear differentiation between respondent rankings for lexicon (dictionary) meaning and lexical (verbal) meaning was evident among the global, North American, and Asian respondents.

**Table 1:** The level of seriousness of the five terminologies across different samples.

Terminology	Ranking of lexical (verbal) meanings					Ranking of lexicon (dictionary) meanings				
	Global	North America	Asia	Europe	Oceania	Global	North America	Asia	Europe	Oceania
Emergency	1	1	1	1	1	1	1	2	1	1
Calamity	2	2	2	1	2	1	1	1	1	1
Disaster	3	2	2	2	2	2	2	2	2	1
Catastrophe	4	3	3	2	2	2	2	2	2	1
Cataclysm	4	3	3	2	2	2	2	2	2	1

The summary of the results in Supplementary E online and “Analysis of perception about natural disaster terminologies” section can be outlined as follows: Firstly, there is a consensus among global respondents regarding the representation of severity order for disaster terminologies in both their lexical (verbal) and lexicon (dictionary) meanings. Similarly, North Americans, Asians, and Europeans share a common perspective on the severity order representation of disaster terminologies for their lexical (verbal) and lexicon (dictionary) meanings. However, for Oceania respondents, agreement is observed only in the lexical (verbal) meaning, not in the lexicon (dictionary) meaning. In essence, an agreed-upon order of seriousness exists rather than random usage of these terms. Secondly, a slight variation exists in the understanding of these terminologies based on the geographical locations of English speakers, particularly in their lexical (verbal) meaning. Nonetheless, such differences are not significant when it comes to the lexicon (dictionary) meaning. In other words, the inclusion of definitions can lead to a general agreement among people, reducing the variance in the severity order representation based on geographical regions. Thirdly, a distinction is evident in perceptions about the order of severity for disaster terminologies between their lexical (verbal) meaning and their lexicon (dictionary) meaning. While a clear differentiation across four severity levels existed for lexical (verbal) meaning, the differentiation was limited to two levels for lexicon (dictionary) meaning. The provided disaster definitions (Oxford Dictionary definitions) did not facilitate differentiation among the disaster terminologies^22,23^. The analysis underscores that these provided definitions did not enhance understanding; rather, they introduced further confusion. Consequently, if these terminologies are to be employed for distinguishing severity levels within a standard classification system, precise definitions for each disaster terminology are imperative.

## Dynamic nature of severity classification

Understanding the usage of disaster terminologies and how global respondents employ them in disaster situations is crucial, particularly when integrating them into a global severity classification system encompassing all types of disasters. In general, it is anticipated that global respondents comprehend and utilize the terminology accurately, and their classifications shift as the severity of an event changes. Within this context, a widespread understanding of disaster terminologies can be inferred. Consequently, these terminologies can be leveraged to delineate severity levels within a global severity classification system, provided that precise definitions are established to enhance people’s comprehension. To examine the hypothesis about how global respondents employ disaster terminologies to convey the severity of an event, a significant event characterized by its diffusion across space and time becomes a more suitable subject for analysis.

The Covid-19 pandemic, which originated in Wuhan, China, in December 2019, swiftly evolved from an endemic to an epidemic, eventually reaching global pandemic status within months. As of March 10, 2023, the pandemic has resulted in over 676.6 million confirmed cases and 6.8 million reported fatalities globally^24^, with new cases reported daily. Covid-19’s far-reaching impact, profoundly affecting various aspects of life worldwide from fatalities to financial crises, makes it a compelling example for this study. To understand public perceptions of major events, an investigation into individuals’ perceptions of the Covid-19 pandemic was conducted.

**Figure 2:**
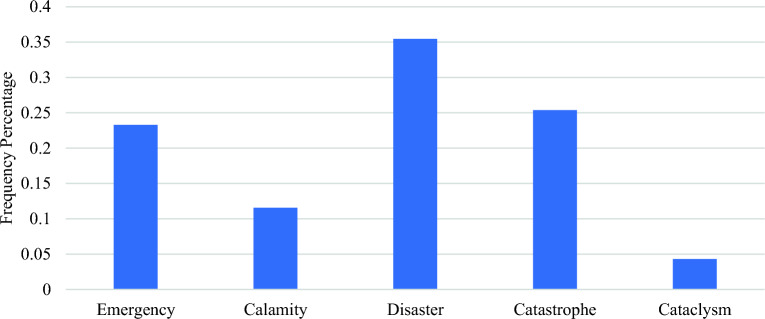
Frequency distribution of people’s perceptions regarding the ongoing Covid-19 pandemic.

During the pandemic, a second survey was conducted to assess respondents’ choice of terminology to describe Covid-19’s severity. Respondents selected a single terminology from five options, with real-time Covid-19 statistics provided alongside. Out of 848 respondents, 674 (79.5%) chose one of the five terminologies to describe Covid-19’s severity. The majority described it as a disaster, followed by catastrophe, and emergency (see Fig. 2).

**Figure 3:**
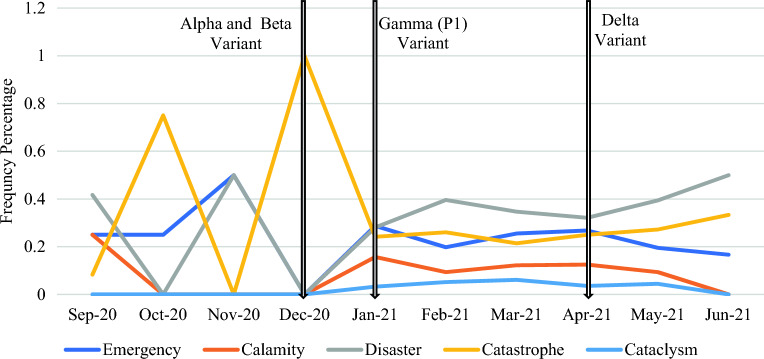
Change in perception about the ongoing Covid-19 pandemic.

Respondents’ perceptions of Covid-19 may have shifted due to its increasing impact. The analysis of respondents’ choices over time revealed that each terminology displayed initial randomness in 2020, followed by a stable pattern emerging in the first half of 2021, and subsequently showed an upward trend for disaster and catastrophe and a downward trend for calamity, cataclysm, and emergency (see Fig. 3).

This shift coincided with the designation of variants of concerns (VOCs) (see Fig. 3 and Table 2). With the designation of Alpha and Beta variants, there was a gradual increase in describing Covid-19 as an emergency, calamity, disaster, and cataclysm, while labeling it as a catastrophe decreased. By January 2021, with the designation of the Gamma variant and total confirmed cases surpassing 100 million, with over two million fatalities, respondents consistently applied a variety of labels to the pandemic. Post-April 2021, as the Delta variant emerged and total confirmed cases surpassed 150 million, with over three million fatalities, the trend shifted towards identifying Covid-19 as a disaster or catastrophe, with a decrease in labeling it as an emergency, calamity, or cataclysm. By the survey’s end in June 2021, 50% of respondents characterized Covid-19 as a disaster, 33.3% as a catastrophe, and the remainder as an emergency; none used calamity or cataclysm. Throughout the survey period, the usage of calamity or cataclysm remained low compared to disaster, catastrophe, and emergency.

**Table 2:** Covid-19 statistics^31^ and designation of variants of concerns^32^.

Date	Global confirmed cases	Global fatalities	Variants of concerns
21 September 2020	33.487 million	1.071 million	
18 December 2020			Alpha and Beta
11 January 2021			Gamma (also known as P1)
25 January 2021	102.574 million	2.333 million	
04 April 2021			Delta
26 April 2021	151.654 million	3.318 million	
28 June 2021	183.282 million	3.981 million	
24 November 2021			Omicron

This case study provides valuable insights into how individuals reference major events and how their perceptions evolve with changing circumstances. As the severity of Covid-19 rapidly increased during the first four months of 2021, reaching over 0.1 to 0.2 billion global confirmed cases and over 2 to 3 million fatalities, people’s choice of terminology became more stable. Their preferences shifted towards terms indicating a higher order of seriousness rather than those with lower levels. As the severity of the pandemic continued to escalate, surpassing 0.2 billion global confirmed cases and 3 million fatalities, people’s usage of terms decreased for those with lower levels of seriousness, while there was an increase in the usage of terms indicating higher levels of severity. This suggests that individuals globally possess a general understanding of disaster terms, and their utilization of these terminologies is guided by their comprehension of the hierarchy of seriousness and events’ severity levels. Therefore, these terminologies can effectively define severity levels within a global classification system, contingent upon establishing precise definitions that enhance people’s comprehension.

## Proposed qualitative universal disaster severity classification

Integrating descriptive terminologies within an emergency management system enhances mutual understanding and simplifies management, minimizing confusion. For instance, using terminologies with escalating severity levels such as ‘emergency,’ ‘disaster,’ and ‘catastrophe’ as descriptive headings aligns with increasing severity, rather than only employing headings like ‘Type 1,’ ‘Type 2,’ or ‘Type 3.’ This approach helps to avoid ambiguity regarding whether Type 1 or Type 5 holds greater significance, as it does for Incident Management Teams Typing (IMTs), a classification used by disaster managers and emergency responders^25,26^. Consequently, a universal linguistic approach that integrates existing severity classification systems becomes imperative. However, the selection of appropriate terminologies for distinct severity levels should be undertaken with meticulous evaluation^15^.

### Proposed sequence of natural disaster terminologies for a global audience

Based on “Analysis of perception about natural disaster terminologies” section and Supplementary C and E online, the order of seriousness for the current dictionary definitions, etymological definitions, and people’s perceptions of natural disaster terminologies is presented in Table 3. This order is being proposed to establish a hierarchy of seriousness for the considered terminologies, tailored specifically for a global audience. As previously mentioned, ‘apocalypse’ is unsuitable for representing severity levels for global audiences due to its religious bias. When determining this order, greater importance was given to the sequence of lexical (verbal) meanings (Column 4 in Table 3) compared to the lexicon (dictionary) meanings (Column 5 in Table 3), as perceived by individuals. This differentiation stems from the fact that the intended order of seriousness is meant for a worldwide audience, where people generally understand a term’s lexical (verbal) meaning without necessarily referring to the provided lexicon (dictionary) meaning. Consequently, the suggested sequence is as follows: emergency, calamity, and disaster for Levels 1, 2, and 3, respectively. However, both catastrophe and cataclysm are placed at the same level based on the convergence of people’s perceptions regarding the lexical (verbal) and lexicon (dictionary) meanings. Nonetheless, when considering the overall mean rank order obtained from respondents’ rankings (as depicted in Fig. 1 and Supplementary Table S6 online), catastrophe and cataclysm are recommended for Levels 4 and 5, respectively. Therefore, based on the analysis of both lexical (verbal) and lexicon (dictionary) meanings, the proposed sequence of the five terminologies from lowest to highest seriousness is as follows: emergency, calamity, disaster, catastrophe, and cataclysm. This arrangement is not arbitrary; it is substantiated by the data and reflects the contemporary viewpoints of individuals on a global scale. Consequently, this sequence is well-suited for a global audience, and these designations effectively function as categories within a comprehensive global severity classification system.

**Table 3:** Terminologies for natural disasters and their changing perceptions.

Level	Etymology	Dictionary	People’s perception		Proposed terminology
			Lexical (without definition)	Lexicon (with definition)	
1	Emergency	Emergency	Emergency	Emergency/Calamity	Emergency
2	Apocalypse	Disaster	Calamity	Disaster/Catastrophe/Cataclysm	Calamity
3	Calamity	Calamity	Disaster		Disaster
4	Cataclysm	Catastrophe	Catastrophe/ Cataclysm		Catastrophe
5	Catastrophe	Cataclysm			Cataclysm
6	Disaster	Apocalypse			

### Proposed qualitative global severity classification system

To establish a universally accepted method of communicating disaster severity levels using a linguistic approach, we have applied the aforementioned proposed order of disaster terminologies to the Qualitative Universal Disaster Severity Classification (QUDSC) developed by Caldera and Wirasinghe^27^, incorporating certain modifications. The selection of QUDSC for this application is primarily attributed to five key factors as described in Supplementary F online.

Table 4 presents Advanced Qualitative Universal Disaster Severity Classification (AQUDSC), a comprehensive system for categorizing all types of natural disasters across stakeholder groups. Five modifications have been introduced to the existing QUDSC. Firstly, the order of seriousness for terminologies has been adjusted, incorporating ‘emergency,’ ‘calamity,’ ‘disaster,’ ‘catastrophe,’ ‘cataclysm,’ and ‘partial or full extinction’ aligning with the general understanding of the global audience as analysed above. Secondly, each level is now assigned a name and definition to create a complete 0–10 level system, including the addition of ‘Emergency Level 1’ to maintain consistency with sub-levels. Thirdly, ‘Type 1’ and ‘Type 2’ terms have been replaced by ‘Level 1’ and ‘Level 2’ to enhance clarity with hierarchical connotation. Fourthly, the definition of ‘emergency’ has been revised to accommodate disasters without human fatalities but substantial damage. Lastly, colour-coding has been adjusted to maintain consistency and aid memorization, with each term assigned a unique color: blue for ‘Emergency,’ green for ‘Calamity,’ yellow for ‘Disaster,’ red for ‘Catastrophe,’ gray for ‘Cataclysm,’ and black for ‘Partial or Full Extinction.’ Lower levels represent light colours, while upper levels represent dark colours. These modifications aim to enhance clarity and facilitate disaster management across all levels (see Supplementary G online for more details).

**Table 4:**
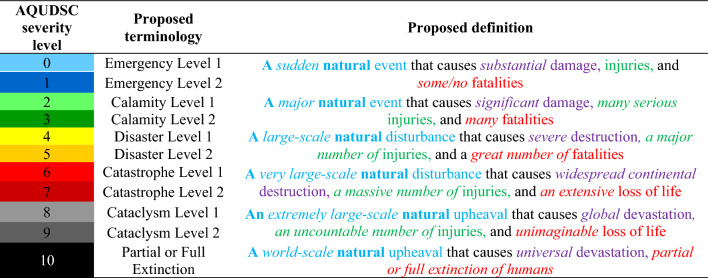
Advanced qualitative universal disaster severity classification (AQUDSC).

The QUDSC was used to create the Initial Universal Disaster Severity Classification (IUDSC)^27^. Subsequently, adjustments were made to the IUDSC to align with the AQUDSC. The resulting Modified Universal Disaster Severity Classification (MUDSC) is presented in Table 5. These modifications have led to improvements to the QUDSC/IUDSC:

- The ranking of disaster terminologies in AQUDSC/MUDSC is suitable for a global audience, as it considers the general understanding and lexical (verbal) meaning of users.
- AQUDSC/MUDSC comprehensively represents the complete range of severity including disasters that lack direct fatalities but cause significant damage to communities, such as the 2016 Fort McMurray fire.
- The system provides a clear labeling strategy to distinguish each level without causing confusion about their respective criticality. Additionally, a consistent colour-coded system facilitates broader communication between the public, emergency services, and media organizations, enabling easy adaptation for any language, country, or culture.

**Table 5:**
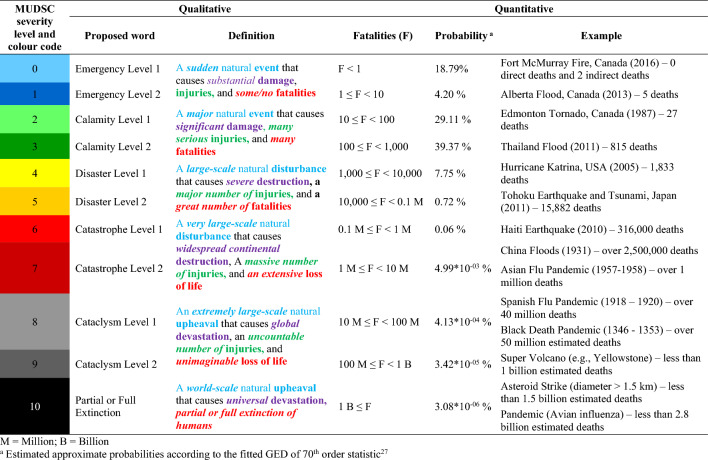
Modified universal disaster severity classification (MUDSC)—fatality-based.

Therefore, AQUDSC/MUDSC enhances the differentiation of disaster severity levels for the global audience, offering a clear understanding of severity along the disaster continuum.

## Discussion

AQUDSC/MUDSC provides standardized terminologies and clear definitions for a global audience to describe the impact of natural disasters. Standardized definitions have significant implications, as outlined in the Technical Report on Hazard Definition and Classification Review 2020^28^. Clear definitions facilitate effective measurement and reporting of risks, thereby contributing to the development of appropriate disaster risk management measures and long-term planning. Standardization supports all aspects of risk management, including multi-hazard risk assessments, warnings and alerts, disaster response and recovery, long-term planning, and public awareness efforts. Furthermore, standardized definitions form the foundation for a uniform database of loss data and information, which makes a valuable contribution to forecasting future events. With consistent, standardized definitions and global-scale risk information, communities at local and national levels can determine the most effective strategies for mitigating the impacts of future events.

MUDSC serves as a global severity classification system for post-event assessment, accommodating various natural events irrespective of the disaster type, location, or occurrence time. It allows for the evolution of severity classifications over time as reports on impacts are updated, aiding responders, and informing public planning and relief efforts. Additionally, it is a comprehensive tool to describe, measure, categorize, compare, assess, rate, and rank the impact of various natural events, ranging from a lightning strike to a super volcanic eruption. Thus, MUDSC simplifies impact assessments, enhances disaster preparedness, and facilitates multi-hazard management by offering a unified classification for regions prone to multiple disasters.

MUDSC enhances warning communications by employing plain language to categorize disasters, ensuring the public comprehends the severity and urgency of evacuation. Plain language communication of warning indications ensures mutual understanding between emergency management systems and the general public. Populations are most sensitive to disasters with high human impacts^7^, and MUDSC explicitly establishes a direct relationship between a disaster and its human impacts. Employing MUDSC for preparedness and mitigation methods, including public awareness campaigns, disaster education, and disaster drills, helps reshape public opinion, capturing the public’s attention and fostering trust and response rates to warnings, minimizing fatalities and injuries during disasters. Its integration into multi-hazard early warning systems contributes to achieving Sendai Framework targets, specifically Target G^29^.

MUDSC enhances disaster preparedness and management globally by providing standardized severity levels. Its implementation is expected to eliminate inconsistencies, facilitate mutual communication among stakeholders, and assess the need for regional, national, and international assistance in managing global disasters. Additional detailed descriptions regarding the significance of the proposed AQUDSC/MUDSC are available in Supplementary H online.

## Conclusion

AQUDSC and its application version, MUDSC, were developed to provide a common language for communicating the severity of natural disasters globally. This system aims to facilitate easier communication and management at all levels by selecting appropriate terminologies and using plain language to describe the magnitude of a disaster’s impact, considering the general public’s perception of disaster terminology.

The main advantage of the MUDSC is its ability to provide a standardized method for comparing natural disasters. It allows for the quantitative and qualitative description, measurement, categorization, comparison, assessment, rating, and ranking of a wide range of natural disasters occurring anywhere in the world. The system covers disasters resulting from various types of events, including those that are diffuse in space and time as well as events with less clear start and endpoints, such as droughts, pollution, and epidemics. It also encompasses conditions that could lead to extinction events or massive phenomena, such as super volcanoes or meteoroid impacts. Furthermore, by facilitating multi-hazards management, disaster risk reduction, and preparedness at all levels and within/across all sectors, MUDSC aligns with the goals of the Sendai Framework.

Importantly, the AQUDSC/MUDSC serves as a common categorization system for all stakeholder groups involved in disaster management and response, including civilians, emergency responders, disaster managers, relief agencies, international/regional/national/local government entities, non-governmental organizations, media, insurance managers/estimators, academics, researchers, and policymakers. By offering a comprehensive view of disaster severity, the system aids in public education, assessment purposes, and decision-making for resource allocation, mitigation, and recovery efforts.

Overall, the AQUDSC/MUDSC is expected to establish a universal standard severity classification system that promotes mutual understanding among different countries’ emergency management systems, eliminates inconsistencies, and provides a common language for describing the impact of disasters worldwide.

### Ethical approval and informed Consents

Informed consent was obtained from all participants before data collection. Consents were granted only for the inclusion of group information in any presentation or publication of results. Please note that the dataset was collected following the Tri-Council Policy Statement: Ethical Conduct for Research Involving Humans 2010 (TCPS 2) and the University of Calgary Guidelines. Ethics approval was granted under certificate ID REB15-0031 and modification ID REB15-0031_MODI for additional questions added to the questionnaire using Covid-19 as a case study. 

## Limitations and future extensions

This ongoing research project aims to develop an advanced multidimensional Universal Disaster Severity Classification System (UDSCS) to comprehensively assess the disaster continuum both qualitatively and quantitatively^61^. The paper introduces an AQUDSC/MUDSC for global comparison of various natural disasters’ impacts. Initially focusing on fatalities alone, MUDSC’s limitation prompted the need for a multidimensional quantitative scale incorporating influential factors like fatalities and damage costs using a disutility function^30^.

The analysis explores the disparity between perceived severity and actual impact, demonstrating the dynamics of community communication. However, the non-random and restricted sample, especially in linguistically diverse regions, may result in deviations in severity perception. Covid-19 serves as an illustrative case study due to its global impact and evolving severity, although perceptions of epidemics and pandemics differ from other disasters. Consequently, there might be disparities in the perception of event severity.

The provided definitions and criteria in AQUDSC offer guidance for adapting the classification to different languages, aiming for equivalence in meaning rather than exact word translations. Future language adaptations may involve proposing suitable terminologies by bilingual experts, followed by surveys to select the most appropriate terms. However, this adaptation process falls beyond the current research scope and remains a potential avenue for future studies.

### Supplementary Information

Supplementary Information.
