# Supplementary material for: Evolution of natural disaster terminologies, with a case study of the covid-19 pandemic
Source: Sci Rep. 2024 Jun 25;14:14616. doi: 10.1038/s41598-024-64736-8 (PMC11199500; doi:10.1038/s41598-024-64736-8)
Supplement: Supplementary file 1 — Supplementary Information. [file 41598_2024_64736_MOESM1_ESM.pdf]

EVOLUTION OF NATURAL DISASTER TERMINOLOGIES, WITH A CASE STUDY OF THE COVID-19 PANDEMIC

H. Jithamala Caldera and S. C. Wirasinghe
[1,2]Department of Civil Engineering, University of Calgary, Calgary, Alberta, Canada.
[1]jhallupa@ucalgary.ca, 0000-0001-8896-7846
[2]wirasing@ucalgary.ca, 0000-0001-5739-1290

SUPPLEMENTARY A: ENGLISH IS THE MOST SUITABLE POINT OF REFERENCE LANGUAGE TO CLASSIFY NATURAL DISASTER SEVERITY GLOBALLY

Currently, there are 7168 languages in use[33]. The top four most spoken languages in the world are English (with over 1.4 billion speakers), Mandarin (with over 1.1 billion speakers), Hindi (with over 0.6 billion speakers), and Spanish (with over 0.5 billion speakers)[33]. English is by far the most spoken language in the world, and the number of English speakers is rapidly increasing[34]. Additionally, English is often referred to as an international language because it serves as the default language in international business, tourism, technology, and much more. Moreover, the top four most widely used official languages in the world, according to the number of countries in which they are used, are English (in 60 countries), French (in 29 countries), Arabic (in 23 countries), and Spanish (in 20 countries)[35]. Furthermore, English is the official language or one of the official working languages in approximately 86 countries, representing almost 45% of the countries in the world. English is also recognized as the official language or one of the official languages by several of the world's most influential organizations and institutions, including the United Nations, the Commonwealth of Nations, the European Union, the African Union, the International Olympic Committee, the North Atlantic Treaty Organization, and the Association of Southeast Asian Nations[36]. Moreover, English is the primary language of communication when dealing with global disasters. In fact, the severity levels of the different classifications used by various stakeholders and the severity levels of the common classifications are generally defined using English, as mentioned below in Supplementary B. Given the predominance of English, for the purpose of this research, the use of English in defining disaster terminology will be the focus of the study.

SUPPLEMENTARY B: EXISTING SEVERITY MEASUREMENT SYSTEMS

Many stakeholders, including first responders, local/regional/international governments, and NGOs, are responsible for managing and reducing disaster risk. When a disaster occurs, they often interact and communicate with one another regarding the severity of an event. Nevertheless, different stakeholder groups currently tend to use different measurement systems; these individual scales have been developed using the linguistic method to categorize the different levels of severity depending on the specific needs of each group. For disaster related medical personnel, de Boer's[1] classification is used, consisting of three levels (accident, calamity, and disaster) depending on casualties and requiring the extra mobilization of medical and other resources. Emergency responders and disaster managers rely on Tierney's[8] severity scale, which includes three levels (emergencies, disasters, and catastrophes) based on the impacts and management challenges associated with response and recovery. Additionally, for the same group, the Encyclopedia of Crisis Management's classification[37] involves four levels (incidents, major incidents, disasters, and catastrophes). Information and database managers use the Munich RE global loss database (NatCatSERVICE) classification, which comprises six levels (small-scale loss event, moderate loss event, severe disaster, major disaster, devastating disaster, and great natural disaster) based on their loss profile and fatalities[38]. Clearly, these individual scales are helpful for their intended groups. However, the terminologies and the criteria used to define different levels by the above classifications differ. These disconnected systems make it even harder to communicate among stakeholders about the severity of a disaster. Consequently, confusion occurs when reporting or communicating the severity of an event. Therefore, developing an agreed-upon terminology for measuring the severity of disasters is important if disaster response is to be effective.

Common classification systems have been developed in the last 15 years for all stakeholder groups to address the lack of uniformity and standardization in describing disaster events. These systems include the following: (1) A Disaster Scope[39] with five levels (small, medium, large, enormous, and gargantuan disasters). (2) Eshghi and Larson's categorization[40] with six severity levels (emergency situation, crisis situation, minor disaster, moderate disaster, major disaster, and catastrophe). (3) A Fatality-based disaster scale[14] with seven levels (emergency, disaster types 1 and 2, catastrophe types 1 and 2, calamity, and cataclysm), which was later updated[22] to include ten severity levels (emergency, disaster types 1 and 2, catastrophe types 1 and 2, calamity types 1 and 2, cataclysm types 1 and 2, and partial or full extinction). (4) The Sendai Framework on Disaster Risk Reduction (SFDRR)[41] with five levels (small-scale, large-scale, frequent and infrequent, slow-onset, and sudden-onset disasters). (5) A Universal Disaster Severity Classification[15] with ten severity levels (emergency, disaster types 1 and 2, calamity types 1 and 2, catastrophe types 1 and 2, cataclysm types 1 and 2, and partial or full extinction). These classification systems provide valuable tools for describing and categorizing various types and levels of disasters.

SUPPLEMENTARY C: DESCRIPTIVE TERMINOLOGIES FOR NATURAL DISASTERS

Emergency, disaster, calamity, catastrophe, cataclysm, and apocalypse are commonly used English terminologies that describe the nature of an event. While these terminologies do exhibit a certain order of seriousness, expressing the precise level of impact is challenging due to several deficiencies in this primary system.

One significant shortcoming involves the interchangeable use of the same terminologies. Within the literature on disasters, an ongoing debate revolves around whether the term 'catastrophe' can be distinguished from 'disaster' or if they are synonyms[37]. In fact, most dictionaries employ these terminologies synonymously[23]. For instance, the Oxford English Dictionary[16] defines a disaster as a catastrophe and then describes catastrophes and calamities as disasters. Similarly, the Merriam-Webster dictionary[42] characterizes a disaster as a calamity and vice versa. The confusion arises due to the broad definitions of disaster terminologies found in standard dictionaries. Additionally, the terminology used by the SFDRR treats 'disaster' and 'emergency' as interchangeable terms. There is no consistent method to differentiate one term from the other, not even in dictionaries or disaster-related sources concerning disaster terminologies[5].

Disaster terminology is not universally understood due to the absence of a consistent method for differentiating between terms[11]. Attaining a shared understanding necessitates a consistent approach to distinguishing disaster terminology and the establishment of a globally accepted standard technique for communicating the severity levels of disasters. A prior comprehension of the evolution and current usage of disaster terminology proves beneficial in formulating a coherent method to differentiate these terms for a global audience. The subsequent sub-sections will delve into this process.

The United Nations Office for Disaster Risk Reduction (UNDRR) is the primary international body responsible for coordinating disaster risk reduction efforts. UNDRR significantly influences all disaster-related initiatives through the Sendai Framework and associated narratives. Within the Sendai Framework for Disaster Risk Reduction (SFDRR), a disaster is defined as "a serious disruption of the functioning of a community or society at any scale due to hazardous events interacting with conditions of exposure, vulnerability, and capacity, leading to one or more of the following: human, material, economic, and environmental losses and impacts"[41]. It is important to note that the effects of a disaster can be immediate and localized but often extend widely and persist for an extended period. Such effects may strain or surpass a community's or society's ability to cope using its internal resources, thereby necessitating assistance from external sources, which might include neighboring jurisdictions or entities at the national or international levels.

The SFDRR also employs the term 'emergency,' which is defined as follows: "Emergency is sometimes used interchangeably with the term 'disaster,' as seen, for example, in the context of biological and technological hazards or health emergencies, which, however, can also relate to hazardous events that do not result in the serious disruption of the functioning of a community or society"[41]. Nevertheless, in the SFDRR, terms such as calamity, catastrophe, or cataclysm are not explicitly defined.

1.1. Evolution of the Lexicon (Dictionary) Meaning of Natural Disaster Terminology Over Time

The linguistic method has been applied to classify various magnitudes of natural disasters that have adversely affected all living beings since the beginning of civilized society. Consequently, these terminologies have evolved over time, and their meanings and levels of gravity have gradually changed. For instance, the term 'disaster' was introduced into the English vocabulary in the late 16th century. It originated from the Italian word 'disastro,' which meant "ill-starred event," implying that the event caused an unfavorable situation or misfortune on the planet. In contrast, its current definition in the Oxford Dictionary describes it as 'a sudden accident or natural catastrophe that causes great damage or loss of life.' Supplementary Table S1 provides the etymological origin and earliest definitions of the natural disaster terminologies: apocalypse, calamity, cataclysm, catastrophe, disaster, and emergency. Supplementary Table S2 presents the current definitions of the same terminologies as documented in the Oxford English Dictionary. A comparison between Supplementary Table S1 and Table S2 highlights the significant shifts in the meanings of these terminologies over time.

The level of seriousness of these terminologies has also changed over time due to the evolution of their meanings. Upon briefly examining the original meanings of these terms, it becomes evident that if we were to rank them, their level of seriousness would differ from today. For instance, the etymological order of these terms, ranked from lowest to highest on a scale index of seriousness, might appear as follows: (1) emergency, (2) apocalypse, (3) calamity, (4) cataclysm, (5) catastrophe, and (6) disaster. However, based on the lexicon (dictionary) meaning

provided by the Oxford English Dictionary, the order of rank on a scale index of seriousness is different: (1) emergency, (2) disaster, (3) calamity, (4) cataclysm, (5) catastrophe, and (6) apocalypse. The lexicon (dictionary) meaning of these terminologies and their order of seriousness vary depending on the era. The fact that the meanings of these words have changed over time highlights the fluidity of the terminology used to describe the severity of natural disasters. This underscores the importance of establishing a common and agreed-upon definition for ranking the seriousness of disasters.

Supplementary Table S1 Etymological definition[27]

Terminology	Meaning	Origin Time
Apocalypse	Uncover, disclose, reveal[43]	Late 14th century
Calamity	Damage, loss, failure, disaster, misfortune, adversity[43]	Early 15th century
Cataclysm	To wash down[44,45,43] (deluge, flood, inundation)[43]	Early 17th century (the 1630s)
Catastrophe	Overturning, sudden turn[44,43] (a sudden end)[43]	Mid 16th century (the 1530s)
Disaster	Ill-started event (the stars are against you)[45]	Late 16th century (the 1590s)
Emergency	To rise out or up (unforeseen occurrence requiring immediate attention)[43]	Mid 17th century (the 1630s)

Supplementary Table S2 Current English dictionary definition[16,27]

Terminology	Meaning
Apocalypse	an event involving destruction or damage on a catastrophic scale.
Calamity	An event causing great and often sudden damage or distress; a disaster.
Cataclysm	A large-scale and violent event in the natural world.
Catastrophe	An event causing great and usually sudden damage or suffering; a disaster.
Disaster	A sudden accident or a natural catastrophe that causes great damage or loss of life.
Emergency	A serious, unexpected, and often dangerous situation requiring immediate action.

1.2. Disaster Lexical Change as per Individual/Situation

The general population's perspective on these disaster terminologies varies. For instance, the term "disaster' is applied to a wide range of situations, from events like earthquakes to instances where two people show up at a party wearing the same clothes. In other words, confusion arises because individuals use these terminologies in diverse ways[46]. Furthermore, these terminologies carry different meanings depending on their contexts, often being used metaphorically with varying connotations[4]. For instance, in Geology, a catastrophe refers to a sudden and violent change in the physical order of things, such as a rapid upheaval, depression, or convulsion affecting the Earth's surface and its inhabitants. Some have even suggested that 'catastrophe' refers to the end of successive geological periods[44]. However, Shakespeare used the term "catastrophe' to express insult: "I'll tickle your catastrophe" in Henry IV, Part 2[47]. Thus, the vocabulary, context, and interpretation of each term are not fixed[48]. Consequently, comprehending the general population's perspectives on these disaster terminologies, along with their potential confusion, misunderstanding, and misinterpretation of the current definitions, is challenging.

The various definitions highlighted above clarify that there are no consistent definitions, methods, or identifiable scales to differentiate these terms from one another. As a result, the same event may be described using several terminologies[15]. An event labeled as a 'disaster' by one observer might be deemed 'catastrophic' or even a 'calamity' by another, depending on personal feelings, knowledge, purpose, and experiences related to the event. On the other hand, events with vastly different levels of severity are often categorized under the same label[27]. For instance, both the 1998 Hurricane Mitch[49] and the 2004 Indian Ocean Tsunami[50] are referred to as catastrophes in various disaster-related sources, including news reports, to indicate the severity of these incidents. However, in comparison, Hurricane Mitch's impact was considerably smaller. It affected eight Caribbean and Central American countries, resulting in 11,000 deaths, while the Indian Ocean tsunami impacted 14 countries across Asia and Africa, causing around 230,000 fatalities. Despite this significant disparity in scale between the two events, the current definitions of these terms do not offer an accurate measurement of disasters' severity.

The meaning and levels of severity of the natural disaster lexicon (dictionary) meaning and lexical (verbal) meaning can vary based on time, individual experience, and the type of situation. Therefore, it is important to investigate whether a specific ordering of these terminologies would be appropriate for labeling the categories of a global severity classification system.

SUPPLEMENTARY D: WILCOXON SIGNED-RANK TEST

If there is agreement among respondents' rankings, we conduct a Wilcoxon signed-rank test for each terminology pair to identify differences and similarities in ranking, revealing people's preferences for these natural disaster terminologies.

H_0: The median difference (M_A -M_B) equals zero. For example, H_0: $M_{Cataclysm}$ -$M_{Calamity}$ =0

A majority of positive signs among the signed ranks (W here W^+ represents the sum of positive ranks and W^- represents the sum of negative ranks) for the pair A-B suggests that respondents tend to rank term A higher than term B, while a majority of negative signs suggest the opposite trend.

Under the null hypothesis, there is an expectation that the numbers of positive and negative signs will be roughly equal, indicating no clear preference. Specifically, within the bounds of random variability, the value of S, which is the sum of W^+ and W^-, is expected to approach zero. As the sample size (n) increases, the sampling distribution of S tends to approximate a normal distribution, denoted as $S \sim N(0, \sigma_S)$, with σ_s calculated as $\sqrt{\frac{n(n+1)(2n+1)}{6}}$ under the null hypothesis, where the mean μ_s is equal to zero.

To account for continuity correction, a value of ± 0.5 is added (subtracting 0.5 when $S > \mu_s$ and adding 0.5 when $S < \mu_s$). The formula for the Z-ratio is $(W \pm 0.5)/\sigma_s$, where the observed test statistic W is calculated as the minimum of $|W^-|$ and W^+. Similarly, when the number of untied pairs n is such that n(n+1)/2 is sufficiently large, a normal approximation can be used. In this case, the mean (μ_w) is equal to $\frac{n(n+1)}{4}$, and the standard deviation (σ_w) calculated as $\sqrt{\frac{n(n+1)(2n+1)}{24}}$. One significant advantage of using permutation tests is that they do not assume a specific theoretical distribution for the test value, making no assumptions about the underlying variable distributions. For example, this test does not require any assumptions about the variable's distribution because z follows a normal distribution. However, this method typically requires a sample size exceeding 60 (Statistics Solutions, 2021). The tests were conducted using IBM SPSS Statistics 26 software. The Wilcoxon signed-rank test is known for its robustness, as it does not require assumptions of multivariate normality or homoscedasticity, making it more reliable than the dependent samples t-test.

A critical value of W is determined to test whether the observed test statistic W supports the null hypothesis (H_0) or an alternative hypothesis (H_a, which can be two-sided or one-sided). If the observed value of W is less than or equal to the critical value, we reject the null hypothesis (H_0) in favor of H_a. Conversely, if the observed value of W exceeds the critical value, H_0 is not rejected. However, to account for multiple comparisons in the Wilcoxon test, it is essential to adjust the significant level. This adjustment prevents an increased likelihood of declaring a result significant when it is not (Type I error). Therefore, we use the Bonferroni-adjusted significance level, which is calculated as $2\alpha/k(k-1)$, where α is the target significance level. This adjustment is particularly important when there are k(k-1)/2 multiple comparisons to be made. In this research case, with k = 5 repeated samples, there are k(k-1)/2 = 10 pairs to be tested. This means that if the p-value is larger than 0.005 for the one-sided test (or 0.0025 for the two-sided test), then the result is not significant for α=0.05.

SUPPLEMENTARY E: SURVEY ANALYSIS

1.3. Lexical (Verbal) Meaning According to a Global Perspective

The first web survey was conducted from August 2015 to December 2020. A total of 1170 responses were collected during this initial survey to assess the rankings of lexical (verbal) meaning. Respondents were not provided with the definitions of the terminologies when they were asked to rank the five terminologies. Among all the respondents, 808 provided rankings for the disaster terminologies. Only 624 respondents (equivalent to 53.3% of the total) completed rankings for all five terminologies in the survey. The first three columns of Supplementary Table S3 (Columns 1 to 3) display the number of respondents who ranked each term and those who did not. A notable number of respondents did not rank 'cataclysm' or 'calamity' in comparison to the other three terminologies. The terminologies 'emergency,' 'disaster,' and 'catastrophe' were more familiar to most people. Furthermore, 'emergency' and 'disaster' were frequently used in a governmental context, while 'catastrophe' was commonly employed in an insurance-related context. The terminologies 'calamity' and 'cataclysm' are typically colloquial. 'Calamity' tends to refer more to emotional reactions[16], whereas 'cataclysm' pertains to flood-related disasters[44,45,43]. Some respondents might not have ranked these terminologies due to their perception of subjectivity rather than unfamiliarity. These respondents might have preferred a more objective differentiation when considering the severity of disasters.

Supplementary Table S3 Number of response rankings collected for natural disaster terminologies

Terminology	N	Missing	Median	Interquartile Range	Mean Rank
Calamity	682	488	3	2-4	2.72
Cataclysm	638	532	5	3-5	3.63
Catastrophe	775	395	4	3-5	3.59
Disaster	800	370	2	2-4	2.95
Emergency	799	371	1	1-3	2.11

1.3.1. Survey sample characteristics

Supplementary Figure S1 Distributions of Survey Sample Characteristics

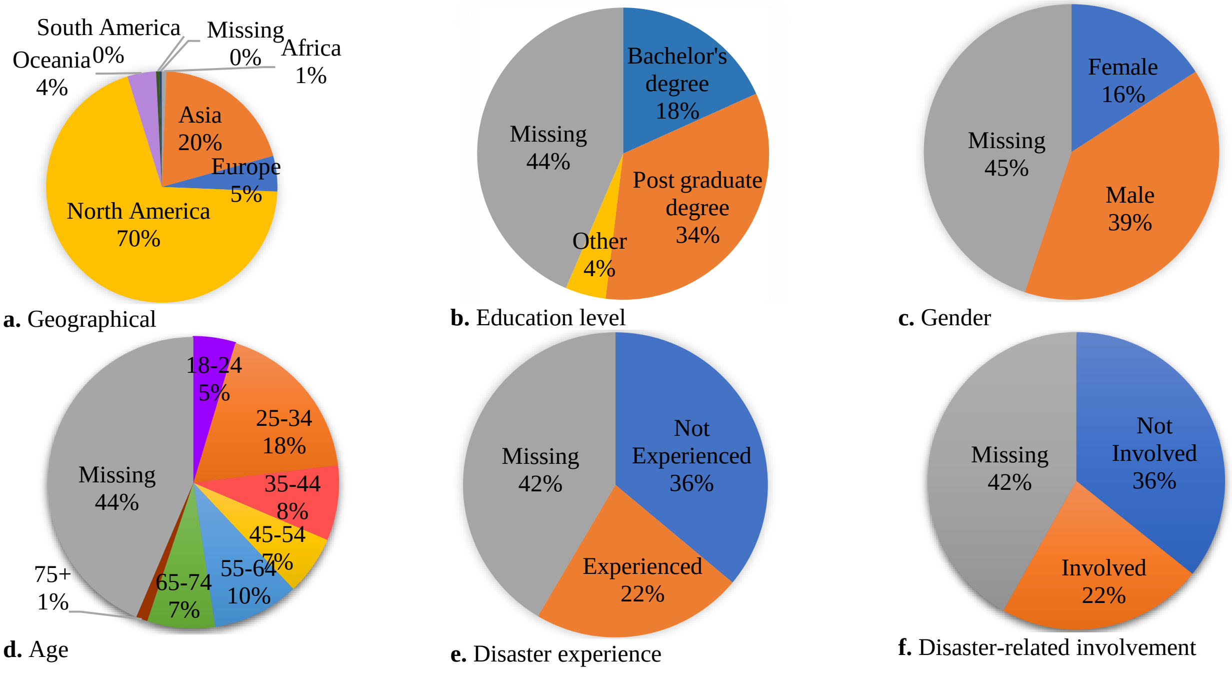

Supplementary Fig. S1 presents the demographics of the 624 respondents who fully ranked all five terminologies in the survey. Supplementary Fig. 1a illustrates the geographical distribution of respondents, revealing that a significant majority (70%) come from North America. The second-highest portion (20%) of respondents originated from Asia, with notable numbers from Europe (5%) and Oceania (4%). A smaller proportion of participants hailed from Africa (1%) and South America (less than 1%). Predominantly, respondents were based in countries like

Australia, Canada, China, Japan, Korea, Singapore, Sri Lanka, the United Kingdom (UK), and the United States of America (USA). Notably, over 75% of respondents hailed from English-speaking nations. Moving on to Supplementary Fig. S1b, it provides insights into the educational levels of the respondents. Among the 56% who responded to the question regarding their education, a substantial 92% held at least a bachelor's degree, constituting 52% of the total respondents. In terms of gender distribution, approximately 71% of the sample identified as male (39% of those who fully ranked all five terminologies and responded to the gender question, as shown in Supplementary Fig. S1c). Supplementary Fig. S1d showcases the age distribution, indicating that the age range of 25-34 holds double the representation compared to other age groups. Regarding experiences related to natural disasters, more than 38% of the respondents had personally experienced such events, and an equivalent proportion had been involved in natural disaster-related work (both comprising over 22% of respondents who fully ranked all five terminologies, as depicted in Supplementary Fig.s S1e and S1f). It is worth noting that there were no discernible differences in terms of education, gender, age, or residence between those who completed the ranking process and those who did not. The results of the non-parametric tests also align with the full sample, encompassing both 1170 and 808 responses.

1.3.2. Respondent ranks for natural disaster terminologies

For respondents who completed the ranking, Fig. 1 displays the frequency of the ranking for the provided terminologies, showing all five terminologies ranked from 1 to 5. The most frequently ranked terminology for the lowest rank one (green bars) is 'emergency,' while 'disaster' takes ranks two (yellow bars) and three (red bars). 'Catastrophe' occupies rank four (purple bars), and 'cataclysm' takes rank five (gray bars). 'Calamity' generated its highest and second-highest frequencies at rank three and rank two, respectively. However, these frequencies were lower than the corresponding frequencies for 'disaster.' The frequency difference between 'disaster' and 'calamity' for rank two (42) was higher compared to rank three (13). Based on the frequency distribution of ranking, the pre-assumption was that 'emergency,' 'disaster,' 'calamity,' 'catastrophe,' and 'cataclysm' might represent the perceived order of seriousness for the five disaster terminologies. Additionally, the median (and interquartile range) perceived order-'emergency,' 'disaster,' 'calamity,' 'catastrophe,' and 'cataclysm'-is shown in Supplementary Table S3 (Columns 4 and 5), aligning with the pre-assumption. In contrast, the mean rank would order the level of seriousness of these terminologies from low to high: 'emergency,' 'calamity,' 'disaster,' 'catastrophe,' and 'cataclysm.' These values are presented in Supplementary Table S3, Column 6, which swaps the ranks of 'disaster' and 'calamity.' To examine the three previously mentioned hypotheses, three non-parametric tests were conducted.

1.3.3. Friedman test

According to the Friedman test, among the 624 respondents who completed the ranking, a statistically significant difference was observed in the perceived order of the disaster terminologies based on their assigned ranks ($\chi^2(4)$ = 402.397, p = 0.000). This indicates that the respondents did not randomly order the five terms, and it can be concluded that global respondents do not consider these terms synonymous when representing disaster severity.

1.3.4. Kendall's test

Kendall's W = 0.161 indicates that there was some agreement among the 624 respondents in ranking the five terminologies. With a sample size larger than 7 ($n > 7$), a considerable and significant agreement existed among the respondents when ranking the different terminologies ($\chi2(4)$ = 402.397, p = 0.000). Thus, it is evident that at least one terminology is ranked significantly higher than the others. Consequently, it can be concluded that a consensus exists among global respondents regarding the perceived order of seriousness of the terms in relation to lexical (verbal) meaning. Therefore, these terms can be considered to represent severity levels on a universal disaster severity scale.

1.3.5. Wilcoxon test

Post hoc analysis using Wilcoxon signed-rank tests was conducted with a Bonferroni correction applied, resulting in a significance level set at $p < 0.005$ (significance values were adjusted to account for multiple comparisons). The first column of Supplementary Table S4 presents the output of the Wilcoxon signed-rank test for each combination pair. Based on the Bonferroni-adjusted significance level, statistically significant differences were observed in perceived ranks for all pairs except the 'catastrophe – cataclysm' pair, in accordance with respondents' rankings (p-values < 0.005, where 0.005 is the Bonferroni-adjusted significance level for ten pairs). However, there were no significant differences in perceived ranks between 'catastrophe' and 'cataclysm' (Z = -0.492, p-value = 0.623 > 0.0025). The order of severity for the natural disaster terminologies-'emergency,' 'calamity,' and 'disaster'-held ranks of 1, 2, and 3, respectively. Both 'catastrophe' and 'cataclysm' received the highest rank of 4. In conclusion, the order

of severity representation for disaster terminology that global respondents agreed upon for lexical (verbal) meaning is: 'emergency,' 'calamity,' 'disaster,' and 'catastrophe'/'cataclysm.' The terms 'cataclysm' and 'catastrophe' are interchangeably used by global respondents to represent the highest severity level.

1.4. Lexical (Verbal) Meaning Across Geographical Areas

This survey focused on terminologies in English used to describe the severity of natural events. English serves as a common means of communication across nations and is internationally the most widely spoken language. It encompasses both native speakers (373 million people with English as their first language) and non-native speakers (1.08 billion people with English as their second/third/etc. language)[33]. Approximately 20% of the global population speaks English[51]. Among English speakers, Asia accounts for 460 million, North America for 350 million, Africa for 237 million, Europe for 212 million, Oceania for over 30 million, and South and Central America for 14 million. The majority of native English speakers hail from North America, predominantly the USA (with 297.4 million) and Canada (with 30 million)[51]. Respondents who ranked the disaster terminologies came from around the world, and their first language may or may not have been English. Therefore, exploring how these terminologies were perceived on different continents becomes intriguing. Additionally, this study delved into whether a significant difference existed in the perception of natural disaster terminologies among English speakers on various continents, considering their lexical (verbal) meaning in comparison to their counterparts on other continents.

Non-parametric statistical tests were conducted to determine whether a significant difference existed in respondent rankings based on their geographical area. Both the Friedman test ($p < 0.05$) and Kendall's W test ($p < 0.05$) demonstrated an overall statistically significant difference in the perceived order of the disaster terminologies, influenced by the assigned ranks to these terminologies. Furthermore, respondents showed some agreement in ranking the five terminologies across Global, North America, Asia, Europe, and Oceania ($p < 0.05$ and $n > 7$). However, due to insufficient data ($n < 7$), the analysis did not include the South American and African continents. The results obtained for pairwise samples and their corresponding p-values are presented in Supplementary Table S4 for respondents who ranked all five terminologies in Global, North America, Asia, Europe, and Oceania.

A Wilcoxon signed-rank test revealed that the pair 'catastrophe – cataclysm' did not lead to a statistically significant change in the perceived severity order across all samples ($p < 0.005$). Similarly, the pair 'calamity – disaster' did not result in a statistically significant change in the perceived severity order for all four continents (North America, Asia, Europe, and Oceania), except for the Global sample. Conversely, the pair 'disaster – cataclysm' yielded statistically significant changes in the perceived severity order for both the Global and North American samples but not for the Asian, European, and Oceania samples. Further analysis revealed that the pairs 'emergency – calamity' and 'disaster – catastrophe' did not induce statistically significant changes in the perceived severity order for the European and Oceania samples. However, the Global, North American, and Asian samples did show such changes for these pairs. Notably, only the Oceania sample exhibited a statistically significant change in the perceived severity order for the 'emergency – disaster' pair. Considering the previous results from the Friedman and Kendall's W tests conducted on Oceania respondents, an overall statistically significant difference was observed in the perceived order of disaster terminologies, with some agreement among respondents in ranking the five terminologies. These results were primarily influenced by the 'emergency – disaster' pair, as Oceania respondents ranked 'disaster' significantly higher than 'emergency.' It is important to note that permutation tests require a sample size greater than 60 for testing significance[52]. Consequently, the European and Oceania continents may not yield reliable results due to insufficient data ($n < 60$).

Based on the analysis above, global respondents ranked disaster terminologies into four levels from lowest to highest: emergency, calamity, disaster, and catastrophe/cataclysm. However, North American respondents employed three levels for the five terminologies: emergency, calamity/disaster, and catastrophe/cataclysm (as indicated in Supplementary Table S5). Similarly, Asian respondents utilized three levels, resembling North American respondents, but the ranking of the term 'disaster' was affected by the complication arising from the 'disaster – cataclysm' pair. Conversely, European respondents applied two levels for the five terminologies: emergency/calamity and disaster/catastrophe/cataclysm. In this case, the ranking of the term 'disaster' was complicated by the 'disaster – calamity' pair. Oceania respondents also adopted two levels for the five terminologies: emergency and calamity/disaster/catastrophe/cataclysm. However, the ranking of the term 'emergency' was further complicated by the results of the pairs 'emergency – calamity,' 'emergency – catastrophe,' and 'emergency – cataclysm.' The results of all three non-parametric tests were consistent with those of the full sample (with 1170 or 808 responses).

Emergency received the lowest rank, and all other terminologies were ranked higher than 'emergency' by Asians, North Americans, and global respondents. Both 'catastrophe' and 'cataclysm' obtained the highest rank among North Americans and global respondents. While global respondents assigned two ranks (Rank 2 and Rank 3) to the 'calamity' and 'disaster' terms, placing them in the middle ranks, North Americans and Asians ranked the two terminologies similarly in the middle. Although the overall results suggest that global respondents generally agree on a four-level representation of severity for the five disaster terminologies, the geographical breakdown reveals variations. North American and Asian respondents agree on three levels, while European and Oceania respondents agree on two levels. As such, a slight discrepancy exists in the perception of the order of seriousness for disaster terminologies among English speakers based on geographical area. Consequently, while there is a consensus on the order of seriousness in the lexical (verbal) meaning of disaster terminology among the global audience, subtle differences arise in understanding these terms according to the geographical locations of English speakers. This raises an intriguing question about potential discrepancies in rankings between lexical (verbal) meaning and lexicon (dictionary) meaning for global audiences. It also prompts exploration into whether rankings of lexicon (dictionary) meaning differ based on respondents' geographical locations. This study further delved into investigating potential significant differences in the rankings of disaster terminologies' lexical (verbal) meaning compared to their lexicon (dictionary) meaning among English speakers.

1.5. Lexical (Verbal) Meaning vs. Lexicon (Dictionary) Meaning

A second survey was conducted to investigate whether a significant difference existed in the perception of the severity order of disaster terminologies between lexical (verbal) meaning and lexicon (dictionary) meaning. In this survey, all five terminologies were presented to each respondent in alphabetical order, along with their definitions from the Oxford English Dictionary (as shown in Supplementary Table S2). Respondents were asked to rank these terminologies based on their perceived level of seriousness, ranging from the lowest (Level 1) to the highest (Level 5). The purpose was to determine whether the introduction of lexicon (dictionary) definitions would alter the general understanding of the terminologies as perceived through their lexical (verbal) meaning. The same analytical approach was applied to assess whether the inclusion of lexicon (dictionary) definitions affected the interpretation of terminologies compared to the initial survey. The responses were collected between September 2020 and June 2021 during the second web survey phase. A total of 847 responses were gathered within that timeframe, with 522 respondents providing rankings for the disaster terminologies. Among these, 443 respondents (equivalent to 52.3%) fully ranked all five terminologies in the survey. This response rate closely resembled that of the first survey, which was 53.3%.

1.5.1. Survey sample characteristics

Out of the 443 respondents who fully ranked all five terminologies in the survey, a significant number were from Asia and North America. There were also a considerable number of respondents from Europe, Oceania, and Africa, but none from South America. The majority of respondents resided in Australia, Canada, China, India, Japan, Nigeria, Portugal, Serbia, Singapore, Spain, Sri Lanka, the UK, and the USA. Notably, over 47% of the respondents were from English-speaking countries.

Of the 69% of respondents who answered the question about their education level, a striking 90% held at least a bachelor's degree, accounting for 62.1% of the 443 respondents. This indicates that the second sample also comprised highly educated individuals. Regarding gender, approximately 49% of this sample were male, constituting 32% of the 443 respondents who answered the gender question. The age distribution of this sample spanned from 18 to 74 years, with the 35-44 group being twice as represented compared to other age groups.

Out of the 72% of respondents who answered the question about their disaster experience, more than 56% had experienced natural disasters, which amounts to over 40% of the 443 respondents. Similarly, out of the 73% of respondents who answered the question about their involvement in disaster-related work, more than 45% had been engaged in natural disaster-related activities, representing more than 33% of the 443 respondents.

It is worth noting that there was no discernable difference in education, gender, age, or residence between those who completed the ranking process and those who did not. Furthermore, the results of the non-parametric tests remained consistent for the full sample, which included 847 or 522 responses.

Supplementary Table S4 Wilcoxon signed-rank test results for pairwise samples of lexical (verbal) meaning

Global (N=624)	North America (N=434)	Asia (N=125)	Europe (N=31)	Oceania (N=25)
Emg. < Cal. (P=0.000)	Emg. < Cal. (P<0.001)	Emg. < Cal. (P=0.001)	Emg. = Cal. (P=0.067)	Emg. = Cal. (P=0.019)
Emg. < Dis. (P=0.000)	Emg. < Dis. (P<0.001)	Emg. < Dis. (P<0.001)	Emg. < Dis. (P<0.001)	Emg. < Dis. (P=0.003)
Emg. < Ctstrp. (P=0.000)	Emg. < Ctstrp. (P<0.001)	Emg. < Ctstrp. (P<0.001)	Emg. < Ctstrp. (P<0.001)	Emg. = Ctstrp. (P=0.007)
Emg. < Ctclsm. (P=0.000)	Emg. < Ctclsm. (P<0.001)	Emg. < Ctclsm. (P<0.001)	Emg. < Ctclsm. (P<0.001)	Emg. = Ctclsm. (P=0.012)
Cal. < Dis. (P=0.003)	Cal. = Dis. (P=0.042)	Cal. = Dis. (P=0.179)	Cal. = Dis. (P=0.038)	Cal. = Dis. (P=0.504)
Cal. < Ctstrp. (P=0.000)	Cal. < Ctstrp. (P<0.001)	Cal. < Ctstrp. (P<0.001)	Cal. < Ctstrp. (P<0.001)	Cal. = Ctstrp. (P=0.126)
Cal. < Ctclsm. (P=0.000)	Cal. < Ctclsm. (P<0.001)	Cal. < Ctclsm. (P<0.001)	Cal. < Ctclsm. (P<0.001)	Cal. = Ctclsm. (P=0.179)
Dis. < Ctstrp. (P=0.000)	Dis. < Ctstrp. (P<0.001)	Dis. < Ctstrp. (P<0.001)	Dis. = Ctstrp. (P=0.008)	Dis. = Ctstrp. (P=0.206)
Dis. < Ctclsm. (P=0.000)	Dis. < Ctclsm. (P<0.001)	Dis. = Ctclsm. (P=0.014)	Dis. = Ctclsm. (P=0.017)	Dis. = Ctclsm. (P=0.526)
Ctstrp. = Ctclsm. (P=0.623)	Ctstrp. = Ctclsm. (P=0.071)	Ctstrp. = Ctclsm. (P=0.048)	Ctstrp. = Ctclsm. (P=0.360)	Ctstrp. = Ctclsm. (P=0.392)

Emg. = Emergency; Cal. = Calamity; Dis. = Disaster; Ctstrp. = Catastrophe; Ctclsm. = Cataclysm

Supplementary Table S5 The seriousness level of the five terminologies across different samples

Terminology	Entire Sample (N= 1170 or 808)	Complete ranking (N=624)				
		Global (N=624)	North America (N=434)	Asia (N=125)	Europe (N=31)	Oceania (N=25)
Emergency	Rank 1	Rank 1	Rank 1	Rank 1	Rank 1	Rank 1
Calamity	Rank 2	Rank 2	Rank 2	Rank 2	Rank 1	Rank 2
Disaster	Rank 3	Rank 3	Rank 2	Rank 2	Rank 2	Rank 2
Catastrophe	Rank 4	Rank 4	Rank 3	Rank 3	Rank 2	Rank 2
Cataclysm	Rank 4	Rank 4	Rank 3	Rank 3	Rank 2	Rank 2

Supplementary Table S6 The mean rank of the samples

Terminology	Lexical (Verbal) Meaning Mean Rank (Order)					Lexicon (Dictionary) Meaning Mean Rank (Order)				
	Global	North America	Asia	Europe	Oceania	Global	North America	Asia	Europe	Oceania
Emergency	2.11 (1)	2.16 (1)	2.15 (1)	1.71 (1)	1.84 (1)	2.64 (1)	2.65 (1)	2.72 (1)	2.23 (1)	2.73 (1)
Calamity	2.72 (2)	2.73 (2)	2.76 (2)	2.35 (2)	2.92 (2)	2.69 (2)	2.73 (2)	2.76 (2)	2.39 (2)	2.73 (1)
Disaster	2.95 (3)	2.92 (3)	2.97 (3)	3.06 (3)	3.20 (3)	3.09 (3)	3.08 (3)	3.01 (3)	3.19 (3)	3.11 (3)
Catastrophe	3.59 (4)	3.53 (4)	3.70 (5)	3.77 (4)	3.64 (5)	3.22 (4)	3.26 (4)	3.22 (4)	3.39 (4)	3.02 (2)
Cataclysm	3.63 (5)	3.67 (5)	3.42 (4)	4.10 (5)	3.40 (4)	3.35 (5)	3.28 (5)	3.29 (5)	3.81 (5)	3.41 (4)

1.5.2. Differences in verbal and dictionary meaning globally and according to the geographical area

Supplementary Table S6 presents a comparison of mean ranks and their corresponding orders for the five terminologies, considering definitions (from the second survey) and without definitions (from the first survey) for the Global, North American, Asian, European, and Oceania samples. When participants were provided with dictionary definitions (lexicon meaning), the mean ranks of the five terminologies, ordered by seriousness from lowest to highest, were emergency, calamity, disaster, catastrophe, and cataclysm for the Global, North American, Asian, and European respondents. However, respondents from Oceania assigned equal mean ranks to emergency and calamity and interchanged the ranks of disaster and catastrophe (refer to Supplementary Table S6, Columns 7 to 11). Similarly, when participants were not provided with definitions and had to rely solely on lexical (verbal) meanings, the mean ranks of the five terminologies in terms of seriousness, from lowest to highest, were emergency, calamity, disaster, catastrophe, and cataclysm for the Global, North American, and European samples. However, the rankings of catastrophe and cataclysm were reversed for the Asian and Oceania samples (refer to Supplementary Table S6, Columns 2 to 6).

The results of the non-parametric statistical tests conducted to determine whether there was a significant difference in respondent rankings among the global samples and their geographical area, both with and without definitions, are presented in Supplementary Table S7. According to the Friedman tests, there was an overall statistically significant difference in the perceived order of the disaster terminologies based on the ranks as assigned to them, with $p < 0.000$ for all samples except Oceania with definitions, where the p-value was 0.215. This indicates that Oceania respondents exhibited random rankings for the five terminologies when dictionary meanings were introduced. While the continuation of the other two tests was not necessary for the Oceania sample, the result is included for consistency. Similarly, Kendall's W tests revealed that there was considerable agreement among the respondents in ranking the five terminologies, with $p < 0.000$ for all samples except Oceania with definitions, which had a p-value of 0.215. Thus, the Friedman and Kendall's W tests collectively demonstrate a significant level of agreement among the respondents when ranking the different terminologies. The results indicated that at least one term was ranked significantly higher than the others in all samples, except for Oceania with definitions, where all samples had a sample size greater than 7.

Supplementary Table S8 displays the outcomes of the Wilcoxon signed-rank test for each pair combination of terminologies among respondents who ranked all five terminologies in the Global, North America, Asia, Europe, and Oceania regions, while considering the provided definitions. The analysis excluded the South American and African continents due to insufficient data. The findings revealed that pairs 'emergency – calamity,' 'disaster – catastrophe,' 'disaster – cataclysm,' and 'catastrophe – cataclysm' did not yield a statistically significant difference in perceived severity order for any of the samples ($p < 0.005$, where 0.005 represents the Bonferroni-adjusted significance level for ten pairs). Furthermore, the 'calamity – disaster' pair did not result in a statistically significant change in the perceived severity ratings for any of the four continents (North America, Asia, Europe, and Oceania), although it did for the Global sample. Notably, the Asian continent exhibited a statistically significant change only in the perceived severity ratings for the 'calamity – cataclysm' and 'calamity – catastrophe' pairs. Additionally, none of the pairs within the Oceania continent generated a statistically significant change in perceived severity ratings, aligning with the results obtained from the Friedman and Kendall's W tests.

Based on the aforementioned analysis, when definitions were provided for the terminologies, respondents generally ranked disaster-related terms into two levels. Specifically, 'emergency' and 'calamity' were perceived as the lowest level, while 'disaster,' 'catastrophe,' and 'cataclysm' were considered the highest level of severity. This two-level ranking was consistent among Global respondents, as well as North American and European respondents. However, the 'disaster – calamity' pair posed a challenge for the ranking of North American and European participants, complicating the position of the term 'disaster.' Respondents from the Asian continent also exhibited a two-level ranking for the terminologies: 'calamity' as the lowest level and 'emergency,' 'disaster,' 'catastrophe,' and 'cataclysm' grouped together at the highest level. Nevertheless, the presence of the 'emergency – calamity' and 'disaster – calamity' pairs introduced complexity to the rankings for 'emergency' and 'disaster.' In contrast, respondents from Oceania showed a lack of distinction among the terminologies based on their definitions, resulting in random rankings. Conversely, similar to the global audience's findings, except for Oceania, where rankings were random, respondents from North America, Asia, and Europe agreed on the representation of two severity levels for the five disaster terminologies in lexicon (dictionary) meaning. This entailed categorizing them as 'emergency/calamity' and 'disaster/catastrophe/cataclysm' for most regions, with the exception of the Asian region that utilized 'calamity' and 'emergency/disaster/catastrophe/cataclysm.' Notably, the provided definitions from the Oxford English Dictionary did not lead to more than two distinct levels of differentiation among the disaster-related terms. In conclusion, in

terms of geographical regions among English speakers, no significant differences were observed in their rankings of disaster terminologies for lexicon (dictionary) meaning, unlike the variation observed in their rankings based on lexical (verbal) meaning.

Supplementary Table S7 The test statistics of the Friedman test and Kendall's W Test

	**Lexical (Verbal) Meaning**					**Lexicon (Dictionary) Meaning**				
	**Global**	**North America**	**Asia**	**Europe**	**Oceania**	**Global**	**North America**	**Asia**	**Europe**	**Oceania**
N	624	434	125	31	25	443	145	181	57	44
Kendall's Coefficient of Concordance	.161	0.152	.145	.389	.196	0.041	0.035	0.027	0.181	0.033
Chi-Square	402.397	263.006	72.320	48.206	19.616	71.975	20.182	19.549	41.277	5.800
Degree of Freedom	4	4	4	4	4	4	4	4	4	4
Friedman test P value	.000	<0.001	<0.001	<0.001	<0.001	0.000	<0.001	0.001	0.000	0.215
Kendall's W Test P value	.000	<0.001	<0.001	<0.001	<0.001	0.000	<0.001	0.001	0.000	0.215

Supplementary Table S8 The results of the Wilcoxon signed-rank test for paired samples with definitions

**Global (N=443)**	**North America (N=145)**	**Asia (N=181)**	**Europe (N=57)**	**Oceania (N=44)**
Emg. = Cal. (P=0.942)	Emg. = Cal. (P=0.718)	Emg. = Cal. (P=0.904)	Emg. = Cal. (P=0.549)	Emg. = Cal. (P=0.897)
Emg. < Dis. (P=0.000)	Emg. < Dis. (P=0.004)	Emg. = Dis. (P=0.085)	Emg. < Dis. (P=0.000)	Emg. = Dis. (P=0.253)
Emg. < Ctstrp. (P=0.000)	Emg. < Ctstrp. (P=0.001)	Emg. = Ctstrp. (P=0.008)	Emg. < Ctstrp. (P=0.001)	Emg. =Ctstrp. (P=0.364)
Emg. < Ctclsm (P=0.000)	Emg. < Ctclsm (P=0.004)	Emg. = Ctclsm (P=0.014)	Emg. < Ctclsm (P=0.000)	Emg. = Ctclsm (P=0.090)
Cal. < Dis. (P=0.000)	Cal. = Dis. (P=0.047)	Cal. = Dis. (P=0.118)	Cal. = Dis. (P=0.010)	Cal. = Dis. (P=0.309)
Cal. < Ctstrp. (P=0.000)	Cal. < Ctstrp. (P=0.002)	Cal. < Ctstrp. (P=0.000)	Cal. < Ctstrp. (P=0.000)	Cal. = Ctstrp. (P=0.249)
Cal. < Ctclsm (P=0.000)	Cal. < Ctclsm (P<0.001)	Cal. < Ctclsm (P=0.000)	Cal. < Ctclsm (P=0.000)	Cal. = Ctclsm (P=0.013)
Dis. = Ctstrp. (P=0.109)	Dis. = Ctstrp. (P=0.189)	Dis. = Ctstrp. (P=0.118)	Dis. = Ctstrp. (P=0.312)	Dis. = Ctstrp. (P=0.743)
Dis. = Ctclsm (P=0.023)	Dis. = Ctclsm (P=0.298)	Dis. = Ctclsm (P=0.107)	Dis. = Ctclsm (P=0.049)	Dis. = Ctclsm (P=0.390)
Ctstrp. = Ctclsm (P=0.129)	Ctstrp. = Ctclsm (P=0.888)	Ctstrp. = Ctclsm (P=0.578)	Ctstrp. = Ctclsm (P=0.042)	Ctstrp. = Ctclsm (P=0.090)

Emg. = Emergency; Cal. = Calamity; Dis. = Disaster; Ctstrp. = Catastrophe; Ctclsm. = Cataclysm

SUPPLEMENTARY F: Qualitative Universal Disaster Severity Classification (QUDSC)

Among all other common severity classifications, QUDSC was chosen to integrate the proposed order of disaster terminologies because it offers the following five key advantages.

Firstly, the QUDSC encompasses a rational and standardized set of severity levels that effectively encapsulates the entire spectrum of disaster severity. It is important to note that determining a uniform number of levels for all disaster types and fields (e.g., medical, rescue, etc.) is impractical due to potential variations based on specific applications or contexts. Nevertheless, mitigating confusion becomes achievable with an appropriate number of levels in place, facilitating a clear distinction between various seriousness categories and thereby establishing consistency. Furthermore, the QUDSC employs a 0-10 level system, coupled with the Universal Disaster Severity Classification Scheme (UDSCS), to encompass a wide range of socio-economic factors (e.g., fatalities, injuries, affected population, cost of damage) that directly correlate with event severity[27]. These factors can be further subdivided into human and damage factors, resulting in 11 levels when employing a logarithmic scale. In Column 2 of Supplementary Table S9, the 11 levels of human factors (H) are outlined, spanning from 0 to 7.95 billion people (the global population in 2022[53]). Similarly, Column 3 of Supplementary Table S9 displays the 11 levels of damage factors (D), ranging from 0 to United States Dollar (USD) 101 trillion (the gross domestic product in 2022[54]).

Supplementary Table S9 Ranges of human and damage factors in 0-10 levels[27]

	Human factors (H)	Damage factors (D)
0	0 = H	0 = D
1	1 < H ≤ 10	1 < D ≤ 10<br>10 < D ≤ 100<br>100 < D ≤ 1,000<br>1,000 < D ≤ 10,000
2	10 < H ≤ 100	10,000 < D ≤ 100,000
3	100 < H ≤ 1,000	100,000 < D ≤ 1 Million
4	1,000 < H ≤ 10,000	1 Million < D ≤ 10 Million
5	10,000 < H ≤ 100,000	10 Million < D ≤ 100 Million
6	100,000 < H ≤ 1 Million	100 Million < D ≤ 1 Billion
7	1 Million < H ≤ 10 Million	1 Billion < D ≤ 10 Billion
8	10 Million < H ≤ 100 Million	10 Billion < D ≤ 100 Billion
9	100 Million < H ≤ 1 Billion	100 Billion < D ≤1 Trillion
10	1 Billion < H	1 Trillion < D ≤10 Trillion<br>10 Trillion < D ≤100 Trillion<br>100 Trillion < D

Secondly, the QUDSC provides a distinct elucidation of the disaster continuum through various approaches: (1) redefining existing terminologies independently, (2) delineating circumstances and impacts, including damage, injuries, and fatalities, and (3) employing descriptive language that effectively captures the order of disaster seriousness. Unlike conventional dictionary definitions, these explanations establish a consistent framework for distinguishing between terms, accurately conveying the true magnitude of distinct severity levels.

Thirdly, the QUDSC is closely linked to quantitative techniques that define clear boundaries and guidelines, thus enhancing its effectiveness in gauging disaster severity.

Fourthly, the integration of the proposed disaster terminologies with the colour-coding scheme of the QUDSC facilitates easy adaptability across languages, countries, and cultures. The colour-coding system proves invaluable for individuals involved in disaster recovery who may be non-literate or unfamiliar with the local language or dialect (particularly in foreign regions). This multi-faceted approach, incorporating definitions, colours, severity level numbers, and quantitative techniques, ensures broader communication among diverse stakeholders and organizations.

Lastly, the QUDSC/UDSCS comprehensively elucidates the disaster continuum by enabling the description, measurement, comparison, assessment, and ranking of the impact of a wide range of natural disasters occurring globally at any time. This system caters to stakeholders seeking to comprehend the severity of various disaster types, both quantitatively and qualitatively.

SUPPLEMENTARY G: PROPOSED MODIFICATIONS TO THE EXISTING QUDSC

Table 4 displays the proposed Advanced Qualitative Universal Disaster Severity Classification (AQUDSC) for all types of natural disasters, encompassing all stakeholder groups, with the introduction of five modifications to the QUDSC.

Firstly, the order of terminologies' seriousness, from lowest to highest, is as follows: Emergency, Calamity, Disaster, Catastrophe, Cataclysm, and Partial or Full Extinction. To address the global audience's general understanding, the seriousness order of 'Calamity' and 'Disaster' has been swapped. As mentioned earlier, during disaster occurrences, there is often no time for reference to clear definitions; people resort to the lexical (verbal) meaning of the term. Hence, the proposed order of seriousness considers both the lexical (verbal) and lexicon (dictionary) meaning analysis in the AQUDSC proposal.

Secondly, each level has been assigned a name and definition to establish a complete 0-10 level system representing the entire disaster continuum. Specifically, 'Emergency Level 1' has been added to the UDSCS 0, also known as 'Emergency.' This inclusion maintains the consistent representation of two levels for the terminologies 'Emergency,' 'Calamity,' 'Disaster,' 'Catastrophe,' and 'Cataclysm' throughout the UDSCS scale.

Thirdly, 'Type 1' and 'Type 2' terminologies for 'Disaster,' 'Calamity,' 'Catastrophe,' and 'Cataclysm' have been replaced by 'Level 1' and 'Level 2.' This change extends to the 'Emergency' terminology, effectively distinguishing the two emergency levels. This modification was necessary to enhance clarity among stakeholder groups by utilizing terminology like 'Level' that inherently implies hierarchy, instead of 'Type,' which lacks such hierarchical connotation. The use of clear language for different categories, including sub-levels, facilitates easier disaster management across all levels.

Fourthly, the definition of 'Emergency' has been revised from "A sudden natural event that causes damage, injuries, and some fatalities" to "A sudden natural event that causes substantial damage, injuries, and some/no fatalities." This alteration accommodates disasters with no human fatalities but substantial damage to communities, like the 2016 Fort McMurray fire. By embracing this change, the AQUDSC scale can encompass events such as wildfires in uninhabited forests, which do not directly impact humans but have lasting effects on ecosystems. Examples include the 2016 Fort McMurray fire (UDSCS 0 – Emergency Level 1) and the 2013 Alberta flood (UDSCS 1 – Emergency Level 2), both record-breaking Canadian disasters.

Finally, UDSCS 0's colour has been changed from 'white' to 'light blue,' the colours for UDSCS 2 and UDSCS 3 have been interchanged, and UDSCS 8's and UDSCS 9's colours have been changed from 'light purple' and 'dark purple' to 'light gray' and 'dark gray.' This adjustment assigns each term a unique colour: blue for 'Emergency,' green for 'Calamity,' yellow for 'Disaster,' red for 'Catastrophe,' gray for 'Cataclysm,' and black for 'Partial or Full Extinction.' Notably, Level 1 terminologies have light colours, while Level 2 terminologies use dark colours. Consistent colour-coding aids memory for the general audience.

SUPPLEMENTARY H: SIGNIFICANCE OF PROPOSED AQUDSC/MUDSC

On most days, more than one natural disaster occurs somewhere in the world, causing immense hardship to living beings and significant property damage and losses. Although these disasters come in all shapes and sizes, ranging from a lightning strike to a super volcanic eruption, their impacts on humans and habitats are quite similar. The Modified Universal Disaster Severity Classification Scheme (MUDSC) shown in Table 5 is proposed to describe, categorize, compare, rate, and rank the total impact of these events and to comprehensively understand the full scope of their severity.

This classification is employed for post-event assessment. The severity classification of an event may evolve as reports on impacts are updated (e.g., the classification for Covid-19 changed with its severity, as demonstrated in Section 5, Fig. 3). As a result, the degree of severity changes over time and with updated reporting on the disaster. For instance, a tsunami, occurring shortly after earthquakes, volcanic eruptions, landslides, etc., could be initially categorized as a 'calamity' based on the severity terminology within the first few hours, depending on reported impacts and casualties. However, the impacts and casualties might escalate in the following days or weeks after the event. Consequently, the severity of the tsunami could be reclassified as a 'disaster' within a day or two after the event, and it might potentially be termed a 'catastrophe' within weeks. Although the accuracy of severity can fluctuate with frequent impact updates, it remains essential to estimate severity even shortly after the event to provide information to emergency responders and for public reporting and planning. For instance, the size of a first-responder contingency depends on the magnitude of the disaster's impact. Hence, predicting severity with a reasonable degree of accuracy can expedite the recovery process. Initial assessments guide crucial decisions such as whether to declare a state of emergency, request international aid, or involve military forces. Other planning determinations encompass storing and delivering resources like food, water, medicine, and clothing to affected areas, setting up temporary medical facilities, determining the scale of these facilities, and establishing the number and duration of temporary housing units. Accurate impact estimation aids in predicting the appropriate allocation of resources and expediting mitigation efforts[38]. Inconsistent disaster identifications could lead to either excessive or insufficient resource allocation for mitigation. Overcompensation may result in significant resource wastage, while under-compensation could exacerbate the disaster's impact. Furthermore, insights gained from past disasters provide valuable information for enhancing communication with emergency services, disaster relief organizations, media, and the general public. A severity scaling system capable of measuring the impact of any natural event will serve as a valuable tool for developing and evaluating national and international disaster planning, mitigation, and hazard reduction strategies[40]. The MUDSC system vividly delineates the scope and total impact of disasters, intended to furnish information to emergency services, disaster relief organizations, media, and the general public. This ensures that public reporting, education, and planning throughout the course of a disaster are as accurate as possible.

The primary advantage of MUDSC is its provision of a single common measurement for all types of natural disasters. In regions prone to multiple disasters (e.g., floods, tornadoes, landslides, etc.), disaster management centers (DMCs) must assess the potential combinations of disasters (e.g., thunderstorms and tornadoes, snowstorms, floods, and hailstorms, or volcanic eruptions and landslides). Subsequently, they determine which combinations are specific to the area under assessment[55]. These assessments involve ranking the most likely individual disaster or combination of disasters that could occur in that area. For example, the Calgary Emergency Management Agency publishes a list of the top 10 hazards and risks in Calgary[56]. After ranking these hazards, DMCs assess the potential impacts of each likely individual event or combination event and make decisions based on their potential combined impacts. These impact assessments, along with their criticality compared to other combinations on the list, enable DMCs to allocate the necessary resources with a justifiable basis. Consequently, MUDSC simplifies impact assessments by offering a unified classification for all types of disasters, which is more informative and consistent than using a variety of unrelated scales for specific disasters.

In addition, warning indications during an event should be conveyed in plain language to ensure that the general public can readily comprehend the severity of an impending disaster and the urgency of evacuation when necessary. In warning communications, a disaster's intensity is often employed to gauge its destructive potential because intensity/magnitude is presumed to be the most meaningful metric for the public. However, intensity and magnitude levels may not be the most effective means to describe the severity of a disaster since they primarily indicate its strength (i.e., hazard potential) rather than its impact (i.e., the vulnerability of a region). The impact of a disaster varies significantly depending on its location, with notable differences between populated cities and rural areas. For instance, a strong tornado may not constitute a significant disaster if it touches down in a remote area with no exposure to human life or property. The event with the highest intensity and magnitude may not necessarily be the most disastrous[15]. It is worth noting that a substantial body of research has presented data indicating that people

often underestimate or disregard warnings for natural disasters and other low-probability events[57,58]. Severe natural disasters are characterized as low-probability, high-consequence events. The MUDSC system employs plain language to categorize disasters; hence, it can be employed to communicate warnings issued by emergency management systems to the general public, ensuring mutual understanding between both parties.

Furthermore, there is currently no global overview of the evidence related to disaster impacts and adaptation, even though the ability to adapt to past disasters is crucial for enhancing preparedness for future events and reducing disaster risk[59]. While natural hazard events offer opportunities for policy changes aimed at enhancing Disaster Risk Reduction (DRR), the frequency and severity of these events, including the number of occurrences, fatalities, economic losses, and affected populations, have not consistently resulted in global improvements in DRR policy[60]. Populations are most sensitive to disasters with high human impacts. Therefore, the MUDSC system should be employed for preparedness and mitigation methods, including warnings, evacuations, public awareness campaigns, disaster education, and disaster drills. These measures can help reshape public opinion regarding the impact of disasters, capturing the public's attention and fostering trust in the techniques utilized by emergency management systems and responders. Consequently, by adopting the proposed terminologies, response times to warnings can be reduced, and response rates can be increased. As a result, public awareness, educational levels, and response rates to warnings can be enhanced using the MUDSC, as it explicitly establishes a direct relationship between a disaster and its human impact. While it is difficult to prevent property damage due to the sudden onset of a natural disaster, proper classifications and terminology, when integrated into an emergency management system, can minimize fatalities and injuries by enabling timely warnings and raising public awareness[7]. Communicating the severity of a natural disaster through the clearly defined terminologies of the MUDSC can facilitate meaningful communication about life-threatening situations, increasing the likelihood of an appropriate public response and promoting public awareness. Additionally, this approach can reduce confusion, enhance mutual understanding between the general public and emergency responders, and improve decision-making capabilities. Nevertheless, these recommended improvements in communication should undergo thorough testing before implementation.

The most significant advantage of the MUDSC is its ability to provide a consistent method for all stakeholders to categorize the severity of various types of disasters. This system incorporates a reasonable and standardized number of severity levels to encompass the entire spectrum of disaster severity. It also establishes a clear hierarchy of seriousness among these levels, accompanied by precise labeling and definitions that convey the severity levels or the potential severity of a disaster. The ascending seriousness levels, ranging from 0 to 10, are defined using qualitative boundaries that consider impact factors such as damage, injuries, and fatalities, and they employ clearly defined descriptive terminologies. This strategic approach eliminates confusion regarding whether UDSCS 1 or UDSCS 10 is the most critical. Moreover, because the MUDSC includes a sensible number of severity levels, events with different degrees of severity are not grouped together. As a result, the MUDSC effectively communicates the scale of a disaster's impact, ultimately reducing confusion and fostering mutual understanding among various stakeholder groups.

Furthermore, during a disaster, when emergency responders communicate with other stakeholders, such as national, regional, and local governments, relief agencies, NGOs, and the media, they lack a standard classification system that offers a unified understanding of the event's severity level[27]. As a result, officials attempting to comprehend the disaster's full impact do not have a consistent scale that can provide a clear understanding of the potential hazard. Consequently, they encounter difficulties in conveying the degree of severity to other stakeholders. The existence of inconsistent and disconnected severity measures means that neither the general public clearly understands the level of emergency, nor do members of emergency management systems have a precise grasp of the potential hazard. The proposed MUDSC system aims to provide a clear assessment of an event's severity for all stakeholder groups. Thus, this system is expected to eliminate inconsistencies and enhance mutual communication among stakeholder groups.

Another advantage of the MUDSC is that it is designed to assess the impact of uncontrollable natural forces, regardless of the type of disaster, its location, or the time it occurs. The effectiveness of a nation's disaster preparedness and management is significantly enhanced when there is mutual understanding and compatibility among countries and various emergency management systems at all levels: international, continental, regional, national, provincial, and local. A government's capacity to manage extreme events can be influenced by the systems they employ. Currently, countries use different systems to manage extreme events. Therefore, there is a need for either a universal understanding of the systems used by other countries or the establishment of a global standard to better prepare for and manage global disasters that impact more than one country. For instance, if a universal system had been in place in 2004 when the Indian Ocean tsunami affected 14 countries in Asia and Africa, it might have

saved thousands of lives. Consequently, the MUDSC is expected to assess the necessity for regional, national, and international assistance.
